# “I Stand Alone.” An Ethnodrama About the (dis)Connections Between a Client and Professionals in a Residential Care Home

**DOI:** 10.1007/s10728-012-0203-6

**Published:** 2012-02-24

**Authors:** Vivianne Baur, Tineke Abma, Ingrid Baart

**Affiliations:** Department of Medical Humanities, VU University Medical Center, Van der Boechorststraat 7, 1081 BT Amsterdam, The Netherland

**Keywords:** Patient involvement, Empowerment, Elderly care, Habermas, Ethnodrama, Transformative research

## Abstract

Client participation in elderly care organizations requires shifting traditional power relations and establishing communicative action that involves the lifeworlds of clients and professionals alike. This article describes a particular form of client participation in which one client was part of a team of professionals in a residential care home. Their joint remit was to plan the implementation of a new personal care file for residents. We describe the interactions within this team through an ethnodrama, based on participant observations and the embodied presence of the researcher (first author). The narratives and voices of all team members are dramatized in this ethnodrama. Throughout the project the team members experienced confusion relating to the confrontation between lifeworld and system, as experienced by the client and professionals in the team. We analyze these tensions by making use of a Habermasian theoretical framework. We conclude that forms for collective client participation in residential care homes should be developed based on communicative action between clients and professionals, with room for emotional engagement.

## Introduction

Resident councils are based on the principle of representative democracy whereby residents’ interests in policymaking and planning in care organizations for older people are voiced. New forms of client participation based on deliberative democracy that create room for the concrete experiences of residents, their lifeworld values and relational empowerment are needed in residential care organizations [6]. From their concrete lifeworld experiences and values, residents can influence and co-produce the way care and services are provided. Trends in client-tailored care and demand-driven care change the culture of health practices. Patients have become clients and are gradually having more voice in care processes [20]. Participation of clients in health care and health research is another development that can be observed [1, 2, 16, 25]. As we envision it, client participation in residential elderly care organizations is about creating partnership relations between clients and professionals, so that enrichment occurs through the amalgamation of various knowledge sources and dimensions of social reality [10]. This means a shift of traditional power relations.

These forms of client participation are explored in the PhD research of the first author, conducted in The Harmony Care Group (pseudonym) a relatively small elderly care organization (five locations) in the Netherlands. Older people who live in these residential care homes are involved in projects to improve care and services in the organization. One of these projects involved the implementation of new personal care files in Rozenberg (pseudonym), one of the locations of The Harmony Care Group. A team of professionals was established to plan the implementation of the new care files in this residential care home. The overall aim of implementing new, more personalized and holistic care files was to improve residents’ involvement with the care they receive. Dialogue between the individual resident and the care worker is an important feature of working with these new personal care files. The researchers (first two authors) advised the management of Rozenberg to involve a resident in the project team as well, so that the client perspective in implementing new personal care files could be taken into account. A client who had shown an interest in the policy planning and organization of the residential home was approached by the head of one of the care units. The first author also participated in the project team, functioning both as a full member of the team (bringing in her expertise on dialogue and participation) and as an academic researcher with an interest in client participation. She followed the developments of this mixed team through participant observation and interviews, and facilitated two evaluation meetings. The research questions that will be answered in this article are: (1) To what extent can communicative action between clients and professionals in an institutional context be realized? (2) To what extent is a Habermasian theoretical framework helpful in order to analyze dynamics of client participation in practice?

This article presents the experiences of the client and professionals with their collaboration in the care improvement team, and reflects on the issues they were confronted with. With this article we aim to analyze these issues by use of a Habermasian theoretical framework. In so doing, we explore how the interrelation between theory and practice can further both dimensions—adding useful insights from practice to theory by which theoretical frameworks are further developed, and, vice versa, adding useful insights from theory to practice in order to create more understanding of issues that are experienced by people in practice. We present an ethnodrama in which confusing moments and situations in the development of the team are described through the narratives and voices of the team members.

An ethnodrama is a script for ethnotheatre, based on research data such as interview transcripts, field notes, journal entries and other artifacts [28, 30]. We choose this form of representing the data because we aim to provide the readers of this article with a vicarious experience, as if watching a stage play or even as if they were part of the project team themselves. By presenting embodied narratives (representing actual situations that arose) we hope that readers will discover the nature of the interactions among the project team members, which needs a thick description of data. We intend to make the readers part of the learning process that occurred in the project team so that they can relate the experiences of the people in our research to their own experiences with client participation (or user involvement in a broader sense). The insights about client participation that follow from the reflections on the ethnodrama and the connection between theory and practice in this article could then be used by others to improve their own practice of client participation. Furthermore, this way of writing also gives us an opportunity to include our personal experiences, narratives and embodied knowledge as researchers and as a team member (first author). We were not distant experts who described and evaluated practice, but we were embedded in this practice and present in our embodied knowledge [15]. This is therefore an ethnodrama as seen through the eyes of the researcher. The words spoken by the “players” are sometimes the literal words as spoken by team members, but most often they are free representations of the opinions, experiences and communication styles of the team members that the researcher encountered (during team meetings, interviews, informal conversations and evaluation meetings). The ethnodrama therefore reflects the outlook of the researcher on this care improvement team. Even though the expressions of the team members in this ethnodrama are represented through the eyes of the researcher, the team members read and approved the research report that forms the basis of this article before publication and they agreed with the way their dynamics and learning process had been represented.

## Methods

### Design

In order to evaluate and support the interactions between the client and professionals in the team for the implementation of personal care files in Rozenberg, we used a combination of qualitative inquiry methods within a transformative theoretical framework. As researchers, we are inspired by the transformative paradigm in research and evaluation [23]. This paradigm focuses on strategies that are culturally appropriate to facilitate understandings in order to create sustainable social change [23]. We are therefore aware, in our work, of the existence of power relations among stakeholders and we explicitly work to balance unequal power relationships, enhance the empowerment of marginalized groups, and create mutual learning processes in organizations as well as in the practice of conducting health research (e.g. [2–4, 6, 7]. The research design was emergent in order to fit the dynamics and issues of the people involved in the project. Choices regarding the design were deliberated on during the project with the members of the team that was to be evaluated. As such, the decision to organize evaluation meetings and to conduct interviews was taken collaboratively with the people from this team who were involved in the project.

### Data Collection

Participant observations were made by the researcher at all team meetings (13 meetings, 26 h in total); she participated in the discussion on the content, took field notes, and spoke informally with the team members before and after the meetings. No predetermined protocol for the observations was used, but attention was given in particular to communication (deliberative quality and language), power relations and emerging values. She also made personal reflection notes in which she reflected on her personal experiences in the team and on her ideas about how the study was proceeding.

Individual interviews were also conducted with five members of the team. The client was interviewed twice: the first time at the start of the project (autumn 2008), and the second time on conclusion of the project (summer 2009). The other team members were interviewed once, around the time the project ended. These interviews had an open conversation style and were guided by the central question “what are/were your experiences with being a member of this care improvement team?” The researcher asked about expectations, feelings of influence and empowerment, interaction with other team members, and how they perceived their own role in the group dynamics. The interviews were recorded and a written summary was handed to the respondents for a member check [17]. All respondents felt that the summaries of the interviews were appropriate, and no changes were made after the member check. Furthermore, at the last evaluation meeting they revealed that the interviews had helped them reflect on their own role in the team and on the interaction and situations that had arisen.

Besides the individual interviews, two evaluation meetings with all team members were organized and facilitated by the researcher. The first evaluation meeting took place after the first half year of the project (spring 2009) and the other half a year after the project had ended (winter 2009). During these meetings, the team members collectively reflected on the way they interacted with each other. Power imbalances were taken into account by the researcher by explicitly giving everyone the opportunity to speak freely. At the last evaluation meeting, the draft version of the research report was the starting point for the team members to reflect on the process as described in the draft report. This meant that the last evaluation meeting served as a joint member check and at the same time it contributed to a further reflection process. During these evaluation meetings, everyone shared positive as well as negative experiences with the group, which led to learning processes within the team.

### Data Analysis and Quality Procedures

The data from the participant observations, interviews and evaluation meetings were analyzed through an iterative process between theory and practice on the one hand, and between researcher and participants on the other. The data from the interviews and the evaluation meetings were thematically analyzed and fed back to the participants for member check, as described above. Further, by writing the research report, the researcher connected the empirical data to theoretical concepts from Habermas’ theory. The draft version of the report was sent to the participants and at the final evaluation meeting this report was the starting point for the discussion. As such, the data described in the final version of the research report were based on a co-analysis process. Several additions to the draft report were suggested by the participants and were approved by them after the researcher had incorporated these suggestions in the report. These additions concerned the observations of the participants that they felt their communication had become more open and on an equal basis *after* the project had ended.

The researcher’s role in this study was to have multiple partiality. This means that the researcher should be open and accessible to all stakeholders, and at the same time be able to take account of existing power imbalances [5, 7]. This is clearly not a neutral stance, but a value-driven approach to research, in which the researcher works for more socially just practices. Taking into account the voice of marginalized groups should therefore also be combined with equal openness to other groups. In practice however, there is a thin line between showing multiple partiality and being seen as an advocate for the group or person in a marginalized or less empowered position. In this research, we saw this difficulty arise because this research project was part of wider PhD research on developing client participation. It was therefore clear that the researcher, who acted as a full member of the care improvement team at that time, was there with the specific aim of supporting the participation of the client in the team. Initially this was not a problem, but it became more complicated when tension between the professionals and the client in the team emerged. However, the evaluation meetings and the interviews brought this issue of the professionals into the open. When it emerged that the professionals in the team started to experience the researcher’s position as an advocate position, the researcher recognized this and explained her position and her wish to have multiple partiality. This way, the reflections on the interactions within the team were also a useful learning process for the researcher and throughout the project she attempted more consciously to find a balance between the parties.

The authors of this article collaborated in analyzing the data. The analysis of the empirical data was first made by the first author (Baur) and the second author (Abma) separately. Joint reflection of the first and second authors on their analysis led to the writing up of the research report. The role of the last author (Baart) was to reflect upon the data from an “outsider” perspective, being a researcher with expertise in disability studies (whereas the first and second authors have expertise in patient participation in elderly care organizations).

### Dramatizing Empirical Data

The way we went about fictionalizing and dramatizing the data into an ethnodrama needs some explanation. According to Saldaña [29], researchers should ask themselves what is the most appropriate and best (“i.e., validly, vividly, and persuasively” p. 61) mode of presentation for qualitative research. In order to write an ethnodrama, the data from field notes, interviews etc. are reduced to what are salient, foreground issues; “the juicy stuff” [29]. Some core aspects that are needed to dramatize qualitative research data are described by Saldaña [29]: participants and characters (possibly including the ethnographer of researcher as character), their words, a plot, monologues and dialogues, and visual action. Therefore, in constructing our ethnodrama, we first pay attention to a description of the characters. The words they speak in the ethnodrama are sometimes literal and sometimes fictionalized words, though always based upon the empirical data. Saldaña [29] compares the writing of an ethnodrama with staging life, with all the boring parts taken out. Therefore, we creatively and strategically edited the stories of the participants to retell them in such a way that a consistent plot (overall structure) developed. This plot however was not just made up by us researchers, but closely related to the overall experiences of the participants as they looked back on the developments of their team during the last reflection meeting. The ethnodrama contains monologue (from the client only, as to emphasize his role in the team) as well as dialogue (representing the interactions between the team members). Further, by constructing the ethnodrama we thought about description of environment, context and non-verbal communication as non-verbal cues reveal much about characters. There are some descriptions in our ethnodrama that refer to visual action, such as facial expressions that show emotions.

## Setting the Scene and Presenting the Players

The Harmony Care Group is an elderly care organization in the Netherlands. This organization financially supports the PhD project of Baur and the Chair Client Participation in Elderly Care that is appointed to Abma. This organization’s motto is: ‘People need people’ and central to their vision is the starting point that care should be tailored to the client. The Harmony Care Group is ambitious in putting this vision into practice and to further improve its care practices it invites researchers, care support services and professional associations into the organization. This also means that the managers in this organization were recruited based on, among other things, their being open to innovation and willing to create a participative and positive climate for clients, volunteers and employees at their locations.

The project team consisted of eight people. They are the characters in our ethnodrama and we are happy to present them to you, using pseudonyms. Mr De Graaf is the client who was asked to participate in this project team. He had been living in Rozenberg for 1 year when he joined the project team. Moniek chaired the project team and she had just started to work as a team leader at one of the Rozenberg care units. Irene, who had recently become a team leader at another Rozenberg care unit, was at that time a “care coach” who coached the care workers to perform their work in better interaction and dialogue with clients. Chantal and Jenny are two care workers with many years’ experience in elderly care. They were assigned to the project team by their team leaders (Moniek and Ans respectively, and the latter not being part of the project team herself). John is an assistant manager at another location of The Harmony Care Group, and he participated in the project team to transfer the outcomes of the project to his own location. Jessica is the quality coordinator of The Harmony Care Group and she is responsible for running projects that involve the quality of care. Vivianne (Baur) is the researcher (PhD student and first author of this article) whose research topic is to explore innovative ways of client participation as partnership relations between clients and professionals in elderly care.

## The Ethnodrama: Scenes of Confusion

We first present three scenes that refer to moments at which the collaboration between the client and professionals in the team caused confusion. After these scenes we will reflect upon the causes and context of this confusion by representing the learning process of this team and linking it to Habermas’ theory on lifeworld and system.

### Scene 1: First Meeting of the Project Team

Mr De Graaf: I’m number 151…

*It is a sunny afternoon, autumn 2008. The members of the “Care Files Improvement Team” are meeting each other for the first time. Present are Jessica, Moniek, Irene, Mr De Graaf, Vivianne, John and Jenny. The meeting starts with an introduction round, since not all members know each other. Moniek and Jessica have already introduced themselves briefly by giving their name and job title. It is now Mr De Graaf’s turn.*

Mr De Graaf: I am number 151. Well, this really is the case. I have a very rare disease, it’s called vasculitis. Have you ever heard of it?

*Some of the others shake their heads, no, they haven’t heard of it. They frown a little and smile, quite a funny introduction this is, they seem to be thinking.*

Mr De Graaf: Well, it’s inflammation of the small blood vessels, and very few people have this disease. I was treated for it last year in hospital. You really should know that I appreciate the important work doctors do. But one doctor made a huge mistake. He gave me the wrong diuretic and I’m still suffering from the consequences. The muscles in my legs have been damaged. I have to walk very slowly and carefully, because my legs sometimes give way. But it’s really important to keep moving, you know. So I take a short walk in the garden every day. Two days ago something happened…I was walking round the garden, and suddenly it went wrong! My right leg stopped working and I fell. I felt so vulnerable. O my God, I thought, now I have to call for help. I wondered if anyone would actually see me and come to help. Fortunately a very kind lady, Mrs. Van Dongen, had already seen me and she came to me straightaway. Some of the girls also came to help me get up again. I was so grateful. Later that day I had a small box of chocolates delivered to Mrs. Van Dongen, to thank her for her kind help and concern. Well, and I happen to know that the Fall Prevention Team is hoping to drastically reduce the number of accidents. Do you know how many people have already fallen this year, here in Rozenberg? No? 150… And a few days ago I became number 151!

*The others are laughing now, because of the humorous, almost triumphant tone of his last sentence. As he was telling his story, everyone sat, listening patiently, nodding now and again to make Mr De Graaf feel he was being heard and understood.*

Moniek: Okay…well, right, thank you Mr De Graaf. I’m glad to hear there were people around to help, and let’s hope you don’t fall again. Shall we get on with our introductions?

Mr De Graaf: Well, actually, I’d like to say something else, if that’s okay?

Moniek *(looking around, frowning a little and glancing at the clock)*: Eh…well, okay, go ahead. But after that let’s move on, because we have a lot of things to get through today.

*Mr De Graaf tells another animated story about another visit to hospital and about how grateful he was for the support of the informal caregiver that accompanied him. He has no children or close family to help him with hospital visits. So he gave the caregiver flowers, even though, officially, it’s against the rules to give volunteers presents. However, Mr De Graaf was very clever, and just told the informal caregiver that these flowers were for his wife. This meant he was not infringing any rules and could still express his gratitude. Then Mr De Graaf puts some newspaper clippings that he’s collected over the years on the table. He tells the others that they are all about innovations in elderly care, and that in the 1970s he was involved in some new ideas about service flats.*

*The introduction round finally moves on, and the others briefly introduce themselves by giving their name and job title. The project team starts to discuss the draft design of the project, drawn up by Jessica and John. It soon becomes clear that there are different expectations about the exact goals of the project, and that the instructions from the organization to the project team are vague. What is also unclear is how client participation can actually be implemented in the project, or how the various different perspectives of the team members relate to one another.*

### Scene 2: Norms and Values

*Winter 2008. At one of the team meetings, Mr De Graaf states that there is an issue that is bothering him a lot.*

Mr De Graaf: I’m very worried about something I saw last week. It was dinner time and I was walking through the hall next to the restaurant. There, right in front of the toilets, I saw Ms Jansen in her wheelchair. All alone, waiting for a care worker to come and help her go to the toilet. You do know how cold it is in that hall, don’t you? And it was a really long time before a care worker got to her. And all that time, Ms Jansen had been sitting there, helpless, outside the toilet. Can’t something be done about this kind of thing?

Moniek: I know Mr De Graaf, you already told me about this yesterday. I appreciate your concern. But I also told you that it’s something we can’t avoid. When people who are having dinner need to go to the bathroom, they can only go with a professional care worker, and not with someone who only works as a volunteer at dinner time. They have to call someone from the team of care workers, and that takes a bit of time.

Mr De Graaf: I thought it was humiliating…O Lord, what if this had been your own mother! It should never have happened! Amazing that something horrible like this can happen nowadays! And then there’s something else that really bothers me: the care workers’ long nails. The patients here are vulnerable! Just like me, long nails could easily hurt me because my skin is so sensitive because of my illness. You should check your care workers’ nails and be very strict with them—if their nails are too long, they should go.

Moniek: This is something that’s in the regulations for care workers, and I really think everyone sticks to these rules quite well.

Mr De Graaf: No they don’t. You should have seen her nails, she scratched my head when she washed me!

Moniek: Okay, I’m sorry to hear that. Please tell me who it was, and I’ll tackle her about it.

### Scene 3: Feeling Excluded

*Spring 2009. The committee is meeting to discuss how the project is going. The atmosphere is tense. Just before the meeting started, Irene and Moniek decided to hold the meeting in a different room from usual. Vivianne went to the old room to pick up Mr De Graaf who was waiting there. He is irritated and tells Vivianne that he did not know that the meeting would take place somewhere else. Vivianne explains that nobody knew that, because it was only decided about five minutes ago. Everyone sits down. Mr De Graaf sighs and fidgets. Chantal and Vivianne notice that he cannot sit in this uncomfortable chair and Chantal gets up to get him another chair. The meeting starts. Moniek and Irene immediately start talking about something the others are unable to follow. Vivianne asks what the aim of the meeting is.*

Moniek: Oh yes, of course. Irene and I put on the agenda that today we should discuss how we can organize working groups of employees, just like we organized conversation groups with clients about the topics in the care files.

Vivianne: Was that what you were to have prepared together with Mr De Graaf? Maybe we have to talk about that first. I understood from him that this didn’t happen.

Mr De Graaf (*annoyed*): No, it certainly didn’t, I never heard anything more about it! I’m never involved in anything, just like I didn’t know that the meeting room had been changed.

Irene: Well, sorry about that, we just decided to change rooms 5 min before we started. And we also already apologized for the way things went with this preparatory meeting. It wasn’t on purpose.

*The meeting continues, chaotically and the atmosphere is tense. Afterwards, Mr De Graaf calls Vivianne and tells her that he wants to stop taking part in the committee. He has the feeling that nothing is ever done about the points he raises and he feels excluded from the professionals.*

### Scene 4: The End of Client Participation?

*Summer 2009. The committee is meeting again to talk about implementing the new care files. There are three special guests: Maria, who works for the organization that is helping elderly care organizations with the implementation; Robert, the manager who is present today to see how the committee is getting on; and Catharina, a nurse who works at Rozenberg. At a certain point, Mr De Graaf expresses his frustration at the organization’s long*-*term planning.*

Mr De Graaf: So what about the need to appoint a caregiver on the work floor who has primary responsibility? When the manager isn’t around, someone else has to be responsible for what happens in the care practice. I already spoke about this a few weeks ago. Do you know why it still hasn’t been arranged?

Robert: Well, I see your point and I think you’re right. But we haven’t decided anything yet about that issue. We first have to go to a conference about this subject. And after that the Board of Directors will have to decide how our organization proceeds with appointing someone with primary responsibility on the work floor.

Mr De Graaf: But how will this problem be solved then? Do *I* have to follow it up? You know, I don’t get the services for nothing, I pay every cent for it!

Robert tells Mr De Graaf that he understands him, but that he cannot change the formal procedure. By the end of the meeting, Mr De Graaf draws attention to the fifteen questions he has written down about the new personal care file.

Mr De Graaf: Can we maybe now have a look at these questions? I’ve written them down for you.

Moniek: I’m afraid there isn’t enough time left. Is it okay by you if we look at it next time?

Mr De Graaf: But I thought we were going to discuss it today. You even came to me with an example so I could look at it from a client perspective, to see if the personal care file would be a useful instrument for the provision of good care. And that’s just what I did.

Irene: It wasn’t the idea of today’s meeting to discuss the content of the care files. Today was meant to discuss future steps with Robert and Maria. You can give me your notes and I’ll type them up, and send them to the others. And then we can discuss these points next time. We do appreciate the fact that you’ve taken a look at the draft version and have written some questions down.

*The discussion seems to be closed, but Mr De Graaf looks very disappointed and emotional. After the meeting, Vivianne and Catharina walk outside with him. They talk with him to try and understand his emotions. It appears that Mr De Graaf had totally different expectations of today’s meeting and he feels excluded. He says that these kinds of things have happened before and that he now wants to quit.*

Mr De Graaf: As a client and as an idealist I stand alone against seven professionals. I don’t get any credit and I don’t have any influence.

## Reflection

This ethnodramatic representation demonstrates the dynamics among the members of this mixed team of care professionals, a client and a researcher. It shows how the interaction between the team members was full of good intentions on the one hand and confusion and frustration on the other. Even though everyone in the team was very aware of the position of the client and the need to give room to his perspective, the client felt unheard and ultimately decided to quit. The two evaluation meetings shed light on the underlying issues that led to this confusion and tension among the team members. When reflecting on the collaboration, the team members spoke about the way they dealt with differing expectations about the project goals, how they communicated with each other, and to what extent everyone felt as though they had influence and worked together as equal partners. We see links here with Habermas’ theory on lifeworld, system and communicative action [18]. In their attempts to collaborate, the client and the professionals in this team demonstrate how lifeworld and system, communicative and strategic rationality, became entangled in a new way, and how this caused confusion for the client and for the professionals.

### Lifeworld and System

Habermas describes how tensions between lifeworld and system have arisen in modern times through processes of rationalization [18]. The system relates to material reproduction in society and is driven by the economy and the state. The system is characterized by instrumental action, directed at profit, the regulation and rationalization of relations between citizens, and the strengthening of one’s own position within the system. People fulfill different, rather circumscribed, social roles in the system, for example as professionals. This social role gives them power in certain domains, they acquire identities that go with such a role, and when communicating they use specific (e.g. professional) rationalities. The lifeworld refers to the symbolic reproduction of society and is characterized by values that are intrinsically cultural and personal, and by communicative action. The lifeworld can be seen as a coherent set of cultural and social norms and identity structures that form the unproblematic horizon for human interaction. Communicative action is directed at finding agreement and shared understanding. Members of a community produce meaning, identity and solidarity by acting communicatively. In an ideal communicative situation (which according to Habermas can never be fully realized) communication proceeds in power free settings, with room for communicative rationalities (which are not in the first place instrumental) and reflexive identities.

Both worlds are intrinsically valuable, and originally they are interdependently connected. However, according to Habermas, system and lifeworld have become uncoupled and problems arise when the system colonizes the lifeworld. This means that the mechanisms of the system penetrate the lifeworld to such an extent that the lifeworld is overshadowed and dominated by system values. Meaning, identity and solidarity become undervalued, leading to alienation, frustration, and unrest.

In elderly care institutions two worlds come together: that of the residents and that of the professionals (care workers and managers). Residents live in the residential care home. They are there day and night, it is the context of their lifeworld. For clients, the actual “here and now” issues are most important to them, since this relates to the lifeworld they represent and in which they are continuously present. It is the task of professionals to ensure that residents feel comfortable in their environment and that they receive the care they need. Professionals thereby enter the living environment and lifeworld of the residents and simultaneously also take with them their own lifeworld: their backgrounds, their own “codes” and personal identity. The question is whether the lifeworlds of clients and professionals meet in communicative action.

In institutions, on the borderline between lifeworld and system, conflict and tension may arise because friction between lifeworld and system becomes visible. This can also be seen in the struggle of resident councils to influence the policymaking process [8]. This state of affairs hampers communicative action between professionals and clients and can lead to a lack of mutual understanding. Communicative action within an organization, such as a residential care home, requires professionals to put themselves in the shoes of the residents, and to open up to the culture, norms and identity of residents. However, when system values and instrumental action prevail and the social roles are therefore dominated by bureaucracy, strategy, legislation and formalities, clients and professionals will experience a gap between themselves.

This is exactly what happened in the team that consisted of a client and professionals. The professionals, who were used to acting instrumentally within the system world of the organization, were confronted with the lifeworld of the client in this team. His personal stories and attempts to discuss with the professionals what he experienced to be core issues of living in a residential care home (such as “what does it mean to be ill”, “what is good care”, etc.), confused the professionals as it was alien to their usual way of communicating and functioning. The client appealed to the lifeworld values of the professionals, to their presence as human beings rather than as bare representatives of the organization they work for. When this did not work out, he first attempted to use system mechanisms himself, for example by urging the professionals to do something about an issue he considered important (see scene 3 in the ethnodrama) by pointing out his power as a consumer (*‘I pay every cent for it!’*). This did not only happen throughout the process, when frustrations had already risen; during the first meeting, described in scene 1, Mr De Graaf already tried to enter the system by presenting his status based on his past merits, i.e. his having been involved in innovative projects for elderly care. He even brought newspaper clippings about these projects to the table in order to reinforce his words. It seemed as though this client wanted to emphasize to the professionals that he was not “only” a client, but a person who had also been involved in innovations and professional projects. In the end he decided to quit because he felt the professionals were not taking him seriously. At the same time, the professionals tried to give the client a place in the system world. This started with the project design in which the project team was set up formally, in the same way as they were used to doing as professionals among themselves. The dominance of the system confused the client: Hadn’t the professionals asked him to join the team in order for him to make a meaningful contribution? Why then did they not listen to him? The client struggled with these questions. During the first period of the project he was still determined to try to break through the barriers of the professionals and to talk about what he considered to be important values for delivering good care. However, as time went on, he became disillusioned, confused about why things weren’t working out, and also frustrated, just like the professionals.

This account raises doubt about the feasibility of communicative action between clients and professionals in the context of institutionalized care. In line with Habermas’ observations on the uncoupling of system and lifeworld, we see here how the system mechanisms thrust themselves between given action situations and their lifeworld horizon [18]. Belderok [14] argues that professionals and clients in elderly care institutions do not share their lifeworld horizons with each other, even though both clients and professionals operate within system and lifeworld. However, clients and professionals participate differently in the system and lifeworld domains. Clients live in the institution where their lives coincide almost fully with the system and lifeworld within the institution, whereas professionals also participate in different domains of system and lifeworld outside the institution. Belderok argues that professionals tend to seclude themselves from the culture, norms and identity of the clients. Moreover, in institutions like these, the system functions separately from the lifeworld because these organizations have become independently functioning domains of financial and bureaucratic power. According to Belderok, the institution manages the persistent scarcity and tries to convince clients that there are simply no more resources available for, for example, creating more jobs for nurses and care workers. If organizations such as residential care homes were to meet the requirements of a communicative community, there would be communicative action among clients and professionals about care and services. Dinner time, activity programs etc. would no longer be defined, organized and planned by the system but also by the norms and culture of the clients’ lifeworld.

However, practice shows that attempts to base policymaking in residential care homes on a more balanced relationship between lifeworld and system is complicated [8]. The dynamics between the members of the team we described in the ethnodrama here, also demonstrate this complexity of attempts to bring the lifeworlds of clients and professionals closer together. Yet the two evaluation meetings created room for all those involved to look at the dynamics from a distance and to learn what was underlying their frustrations and what they could do about it. Through the reflection moments, this team shed light on the different validity claims and experiences of the members. The reflection meetings meant searching for communicative action in a context that is characterized by the differing lifeworlds of clients and professionals and the dominance of system.

### Searching for Communicative Action: Acknowledging Differences First

The reflections of the team members during the evaluation meetings resulted in a discussion about differences in *power*, *identity* and *rationality*. By reflecting together on the dynamics of the team, the team members learned in the first place that there were differences between them and that these differences related to their identity, power and the rationalities they used in how they communicated. By the end of the project, the team started to realize that the problem had not been the existence of these differences but the fact that these differences had not been recognized most of the time. John commented on this during the last evaluation meeting, after the project had ended:

*This has been a pitfall. I thought everyone is equal and so I didn’t want to distinguish between people. Now we have learned that differences need more attention.*

In the section below we present the issues of power, identity and rationality in more detail. Through reflection on group dynamics, the team members learned that communication between them was hampered because they did not yet recognize the existing differences in power, identity and rationality. The team members concluded that for new projects in which professionals were going to work together with clients, some of the pitfalls they experienced can be prevented by: (1) starting the project by getting to know each other on a more personal level (for example organizing some social event); (2) making room for evaluation of the interpersonal relations on a regular basis, and (3) exploring mutual expectations, respecting differences and designing the project collaboratively. Even more importantly, their overall conclusion was that making mistakes is not such a bad thing, since it leads to learning processes if all those involved engage in evaluation and deliberation.

#### Power

The team members experienced that they were in different power positions with respect to each other. The client left the team ultimately because he felt he was not taken seriously. For him, it was not enough that the professionals listened to his stories. They did not deliberate with him about his message and he did not see any actions taken in practice as a result of his urgent calls upon the professionals. This made him feel powerless, without any authority or power to break through the barriers he experienced in-between himself and the professionals. Also the professionals saw themselves confronted with their hierarchical power position. They experienced a tension between their care-taking responsibility (wanting to protect clients) and this new way of collaborating with clients. This resulted in the professionals being very cautious not to offend Mr De Graaf and thus not speaking up about their frustrations or about their differing opinions and perspectives. Professionals acknowledged during the last reflection meeting that they had been so pre-occupied with the idea that they had to protect clients and that they should do everything to prevent clients from being hurt, that they did not communicate openly with Mr De Graaf. His sensitivity and emotionality even increased their sense of withholding themselves from being too up-front and open with him. This discrepancy between on the one hand wanting to protect a client from negative experiences/being hurt and trying to work together as equal partners in a team led to the actual reinforcement of the existing unequal power relations between caring and responsible professionals and vulnerable clients in need of protection. Both the client and the professionals in this team thus experienced that differences of power position played a significant role in their interactions. The more the client tried to urge the professionals towards understanding and action (thus trying to gain more power and voice), the more they felt the need to protect him from feeling too responsible, but also to protect him from their frustrations about his way of communicating and their own limited power to actively change the status quo in the organization with regards to the practice improvements this client asked for.

#### Identity

The professionals in the team participated on the basis of their task in the organization. They played the role they were used to playing. However, the client did not have a formal function from which to derive his role and identity within the team. He could only be present as a human being, with his own character and communication style. The professionals found this confusing, particularly because this client challenged them continuously to become personal, to share their own opinions and experiences, and to discuss ethical issues that could also have been part of their own personal lives. Professionals felt that most of the subjects the client brought into their discussions were valuable and gave a good insight into the practice of care from a client perspective. However, the range of issues the client wanted to discuss was much broader than the scope and aims of the project team with the mission to implement new personal care files. Even though the professionals gave room to the stories of the client, the client felt they were not able to place themselves in his shoes and that they did not know what it means to be old. On the other hand, the professionals felt that the client did not understand their identity and position. Personally, they might agree with him and understand him, but in their professional roles they did not always feel room to be open about their personal feelings, ideas and experiences. Looking back at the project, one of the professionals said she realized now that all of the team members were ‘hiding safely behind their professional role in the team’, not showing the person who they really are. At the same time, the client was trying hard to get through this professional outlook of the team members and to get them to reflect on issues from the basis of their own personality and character. This caused tension.

#### Rationality

In this team, the preponderant strategic rationality of the professionals clashed with the input of a more communicative rationality on the part of the client. The client’s aim was to contribute to good quality of care for himself and the other clients, and deliberation about ethical issues and his own experiences formed the core of the rationality that guided his actions and communication. The professionals however, were used to a certain style of communication and being in a meeting which did not allow much space for personal stories and value-laden discussion. By the last evaluation meeting, they discovered that they were not used to making expectations explicit at the start of a project. This also did not happen at the start of this team, whereas there were different opinions about the goals and processes of the project. This led to the continuous repeating of discussions that did not get to the core of the problem: differing expectations that had not been given room to be expressed and explored together.

## Discussion and Conclusion

In this article we want to answer the questions ‘to what extent can communicative action between clients and professionals be realized?’ and ‘to what extent is a Habermasian theoretical framework helpful in order to analyze dynamics of client participation in practice?’. This project enabled us to discover that acknowledging differences between people (clients and professionals in this case) is the first step towards communicative action. By engaging in reflection and deliberation, the team members learned that issues of power and identity were underlying and hampering the communication process between them. However, acknowledging differences and then trying to build rational reflection and deliberation does not automatically lead to communicative action. One could say that the client and the professionals in our case project engaged in communicative action during the reflection moments. They rationally reflected upon their interactions, guided by a facilitator (first author) who paid attention to creating an ‘ideal speech situation’, encouraging participants to be open and sincere. However, the question arises whether this ideal speech situation could really be reached and, moreover, if the Habermasian definition of this ideal situation for deliberation, with its focus on rational arguments, is appropriate. Feminist and political scholars criticize the rationalistic model of deliberation envisioned by Habermas, because it excludes emotive language and emotional engagement [11, 19, 22, 32]. They highlight the importance of emotional engagement and storytelling to the vitality and validity of deliberations, for example in public participation forums [11, 22]. As such, deliberative spaces can even be considered as emotional spaces: ‘spaces in which identities are negotiated, constructed and possibly transformed, righteous anger, pain and frustration are expressed, and hopes and aspirations are pursued’ [11]. As Barnes further explains, ‘emotional expressions emphasize the significance of the issues that are the substance of debate and the particularity of the situations that demand a response’ [11].

If we take this broader concept of deliberation and the role of emotions to consider the dynamics of the team in our project, we see that the emotional engagement of the client was met with insufficient dialogical understanding by the professionals. Although they told the client that they valued his input, they did not really relate to his emotional expressions, nor did they share their own emotional reactions in the team. Overall, one could conclude that the professionals did not recognize this importance of the emotional expressions of the client since they were not used to this specific form of ‘rationality’. As a consequence of the disregarding and ruling out of the clients’ contributions in the team on the basis that these were not expressed in the right way and about the right topics, a lack of reciprocal trust existed. It is argued that reciprocal trust is required for effective participation to take place [13]. In the light of these insights about the importance of emotional engagement and expression for communicative action, effective participation and deliberation to be realized, we can conclude that one of the reasons the collaboration between the client and professionals turned out to be unsatisfactory for all parties, is due to the lack of acknowledgment of the importance and validity of the emotional expressions of the client. Moreover, the professionals felt not capable or willing to engage in emotional expressions themselves as well. This would not fit with their identity, rationality and power position within the system in which they played their role. This hampered the deliberative process to flourish. We thus argue that real communicative action between clients and professionals can only occur if they are all open about their emotions, share their frustrations and engage in storytelling and the discovery of shared experiences and values.

Furthermore, we argue that this project shows the practical inadequacy of rational argumentation as the supposed core of deliberative practice. This has implications for the extent to which the Habermasian framework on communicative action and deliberation is helpful in analyzing the dynamics of client participation in practice. It is useful in order to discover the tensions between system and lifeworld: it can be argued that the professionals did not engage in emotional expressions, even though they felt emotions and frustrations, because they were so much part of the system rationality that they could not free themselves from it. Further, using Habermasian theory to explain what happened in practice was useful input for the learning process of those involved in the project. However, we also see limitations to the applicability of the Habermasian framework to explore the dynamics of the collaboration of clients and professional, because it offers only a limited view on how deliberation takes place and excludes emotional expressions as valuable contributions to deliberation. As such, we would like to propose to broaden the Habermasian idea of deliberative quality in communicative action with the value of emotional expressions, in line with the arguments of feminist and political scholars on public deliberation (*e.g.* [11, 22].

Another question that we want to address is to what extent it is possible to create communicative action between clients and professionals. After all, the client withdrew from the team, and the real learning process only occurred once the project had ended, when interaction had become less emotionally charged. It appears that communicative action between the professionals and client was only possible beyond the professional goals and the dominance of system logics. And still, the emotional engagement of all participants was not being acknowledged as a valuable prerequisite for communicative action and as a valid form of argumentation. Thus, we argue that the development of partnership relations between clients and professionals in health care institutions can only be achieved if power relations and differences in identities and rationalities are taken into account, as well as different forms of expressions, including emotional expressions and storytelling. To this end, clients should first be supported in developing empowerment and creating space for their own forms of expressions and experiences before going into dialogue with professionals. In this study, Mr De Graaf was an individual who felt he had to stand up to the system on his own (*“As a client and as an idealist I stand alone against seven professionals.”*). Other studies have shown the impact of bringing people in a marginalized position together to develop their own voice and empowerment [6, 7, 9, 12, 24, 26, 27]. In order to establish communicative action between clients and professionals, attention should be given to the process of participation: a process that should start by recognizing differences of power, identity and rationality, and by bringing clients together for social support, storytelling, sharing experiences and emotions, and developing empowerment.

The urge to establish collaboration and partnership between clients/patients and professionals is not only apparent in the context of residential care homes. In recent years client participation and involvement has become a goal of government health care policy. The idea behind this policy is that the democratic involvement of patients and consumers in health care is in itself merited, and also that input from the patients’ lifeworld can lead to better care. However, in many instances of involvement, system logics come to dominate the way involvement is organized; in order to be involved, patients and clients adapt to professional ways of working while neither the differences in power and identity nor professional rationalities are discussed [21]. Therefore, also in other attempts by professionals to work together with patients, communicative action is difficult to bring into being.

Communicative action can only exist when empowered clients speak with professionals about their lifeworld values. Openness and a willingness to change are required of professionals if these dialogues are to succeed. We believe that this open attitude is not always to be found in practice among professionals and for a variety of reasons: professional rationality, workload, cutbacks, staff shortage, personal character etc. Attention should therefore be paid to giving professionals room for deliberation with each other about the underlying values and issues that accompany their work. Empowerment is always relational [31] and therefore client participation, and partnership between clients and professionals, require the empowerment and learning of all parties involved. Based on these assumptions and lessons from practice and Habermasian theory, we developed the PARTNER intervention, which stands for: Participation, Action, Relations, Trust, Negotiation, Empowerment and Responsiveness [8–10]. The PARTNER intervention aims at empowerment and partnership, and sets out guidelines for patient participation under the guidance of a facilitator. The intervention assumes that participation requires, first of all, deliberation in the context of converging interests in order to empower marginalized groups. This intervention supports clients and professionals in the process of building partnership relations and communicative action. The core of this intervention is that clients and professionals engage in lifeworld dialogues within their ‘own’ group first, before engaging in dialogue with each other and developing a joint agenda for practice improvements. The PARTNER intervention creates room for involvement of clients/patients and professionals on the basis of developing mutual understanding, communicative action and lifeworld values. By taking an appreciative approach, the PARTNER intervention encourages clients and professionals to think and act “out of the box”; out of the assumed impossibilities and restrictive system rationality. Clients and professionals who have worked together with the PARTNER intervention describe feelings of relational empowerment and partnership relations [9]. However, questions still arise as to how these acquirements can be anchored in the culture change (routines, thinking and behavior) of all those involved in the health care organization (which involves clients, professionals and even volunteers and family members). We therefore conclude that communicative action between professionals and clients in health care, however complicated, can actually be reached. However, more academic research and practical knowledge is needed to answer the important question of how communicative action between professionals and clients/patients, once developed (taking into account differences of power, identity and rationality), can be sustained in the long term.
